# Exercise-Induced Hypoalgesia: Cellular and Molecular Mechanisms Linking Pain Modulation and Stress Regulation—A Narrative Review

**DOI:** 10.3390/cells15100858

**Published:** 2026-05-08

**Authors:** Pavle Gajic, Ivana Kovac, Graham Lubinsky, Nebojsa Nick Knezevic

**Affiliations:** 1 Department of Anesthesiology, Advocate Illinois Masonic Medical Center, Chicago, IL 60657, USA; mercurial.pg@gmail.com (P.G.); kovac.ivana29@gmail.com (I.K.); graham.lubinsky@aah.org (G.L.); 2 Department of Anesthesiology, School of Medicine, Wake Forest University, Winston-Salem, NC 27157, USA; 3 Department of Anesthesiology, University of Illinois, Chicago, IL 60612, USA; 4 Department of Surgery, University of Illinois, Chicago, IL 60612, USA

**Keywords:** exercise-induced hypoalgesia, chronic pain, stress regulation, descending pain modulation, endocannabinoid system, myokines, neuroinflammation, exercise therapy

## Abstract

Exercise-induced hypoalgesia (EIH) illustrates how physical activity can reshape the biology of pain while simultaneously influencing the systems that regulate stress. Acute and repeated exercise can reduce pain sensitivity in healthy individuals and in some chronic pain populations, yet the magnitude and consistency of these effects vary substantially across individuals, diagnoses, and exercise protocols. This variability suggests that EIH is not a uniform response, but an adaptive multisystem process shaped by neural, immune, endocrine, metabolic, musculoskeletal, and psychosocial factors. This narrative review synthesizes evidence linking pain modulation and stress regulation across biological scales. Exercise engages descending pain modulatory circuits involving the periaqueductal gray, rostral ventromedial medulla, and spinal dorsal horn, while also influencing endogenous opioid, endocannabinoid, serotonergic, and noradrenergic signaling. These pathways are relevant not only to nociceptive inhibition, but also to affective regulation, hypothalamic–pituitary–adrenal axis activity, autonomic balance, and perceived stress. In parallel, exercise-related neuroimmune changes, including modulation of microglial activity, cytokine signaling, and myokine release from skeletal muscle, may connect peripheral metabolic activity with central mechanisms of pain and stress adaptation. Importantly, the evidence supporting these mechanisms differs in strength: some findings derive from human experimental and clinical studies, whereas others are supported mainly by preclinical or translational research. By distinguishing direct evidence from mechanistic inference, this review highlights how exercise may support hypoalgesia, stress resilience, and functional recovery, while also emphasizing the need for biomarker-informed, personalized exercise strategies in chronic pain management.

## 1. Introduction

Chronic pain represents a major global health burden and is increasingly recognized as a multidimensional condition shaped by interactions between nociceptive processing, neuroimmune signaling, and psychosocial factors. In addition to persistent nociceptive input, many chronic pain states involve maladaptive neuroplasticity within the central nervous system, including impaired descending inhibition, spinal hyperexcitability, and neuroinflammatory activation of glial cells [1,2,3,4]. These alterations contribute to central sensitization and to the persistence of pain beyond the resolution of peripheral tissue injury. Importantly, chronic pain frequently co-occurs with heightened psychological stress, anxiety, and mood disorders, suggesting that overlapping neural and neuroendocrine mechanisms regulate both nociceptive and stress-related processes [1,2,3,4,5,6,7,8].

Regular physical exercise has emerged as a clinically effective and mechanistically intriguing intervention capable of influencing these interconnected systems. A large body of experimental and clinical research demonstrates that both acute and repeated exercise can produce reductions in pain sensitivity, a phenomenon known as exercise-induced hypoalgesia (EIH) [2,9,10]. EIH is commonly observed as increases in pressure pain thresholds, reductions in temporal summation, and improvements in endogenous pain inhibition following physical activity. Although initially described primarily in healthy individuals, similar effects have been documented across multiple clinical pain populations, including chronic musculoskeletal disorders [1,2,10]. In parallel, exercise is widely recognized for its beneficial effects on psychological stress, anxiety, and depressive symptoms, indicating that shared biological mechanisms may underlie the analgesic and stress-modulating effects of physical activity [5,6,7].

At the neurobiological level, exercise engages multiple interconnected regulatory systems that operate across molecular, cellular, and network scales. These include activation of descending inhibitory pathways originating in the periaqueductal gray and rostral ventromedial medulla, modulation of endogenous opioid and endocannabinoid signaling, regulation of monoaminergic neurotransmission, and suppression of neuroimmune activation within spinal and supraspinal circuits [3,4,9,11,12,13]. Concurrently, skeletal muscle contraction triggers the release of myokines that exert systemic anti-inflammatory and neurotrophic effects, providing a mechanistic link between peripheral metabolic activity and central neural adaptations [14,15,16,17,18]. Exercise also influences hypothalamic–pituitary–adrenal axis regulation, autonomic balance, and musculoskeletal tissue resilience, all of which contribute to the broader physiological context in which pain perception and stress responsivity are regulated [6,7,8,19,20,21,22,23,24].

Despite the growing body of literature on exercise-induced hypoalgesia, the underlying mechanisms remain complex and are distributed across multiple biological levels. Rather than focusing on resolving specific unanswered questions, this review aims to integrate current knowledge on neural, neuroimmune, and musculoskeletal mechanisms and to highlight how these processes collectively contribute to both pain modulation and stress regulation. Particular emphasis is placed on identifying mechanistic connections and translational implications across different levels of biological organization [1,2,3,4,5,6,7,8,10]. This approach is intended to provide a conceptual framework rather than a comprehensive or quantitative synthesis of all available evidence. Understanding how these diverse mechanisms interact across biological scales is essential for developing more precise and individualized exercise-based strategies for chronic pain management.

The aim of this review is to provide an integrative overview of the cellular, molecular, and systems-level mechanisms through which physical exercise modulates pain and stress. We first examine the neurobiological foundations of exercise-induced hypoalgesia, focusing on descending pain inhibitory circuits and endogenous neuromodulatory systems. We then discuss the role of neuroimmune interactions and inflammatory signaling in shaping pain sensitivity and stress responses. Finally, we explore musculoskeletal adaptations and systemic physiological mechanisms that contribute to the broader therapeutic effects of physical exercise in chronic pain conditions.

### Narrative Review Methodology

This narrative review was designed to synthesize mechanistically relevant evidence on exercise-induced hypoalgesia, chronic pain, and stress regulation. A structured literature search was conducted using PubMed, Google Scholar, Scopus, and Web of Science. Search terms included combinations of “exercise-induced hypoalgesia”, “exercise analgesia”, “exercise and pain modulation”, “chronic pain and exercise”, “stress regulation and exercise”, “descending pain modulation”, “endogenous opioid system”, “endocannabinoid system”, “serotonergic pain modulation”, “noradrenergic pain modulation”, “neuroinflammation”, “microglia”, “myokines”, “skeletal muscle endocrine function”, “exercise therapy”, “fibromyalgia”, “chronic low back pain”, “knee osteoarthritis”, “whiplash-associated disorders”, and “kinesiophobia”. Boolean operators were used to combine concepts related to exercise, pain, stress, and molecular mechanisms. Representative search strings included: (“exercise-induced hypoalgesia” OR “exercise analgesia” OR “exercise and pain modulation”) AND (“pain” OR “nociception” OR “chronic pain”) AND (“stress” OR “stress regulation” OR “HPA axis” OR “anxiety”) AND (“endocannabinoid system” OR “opioid system” OR “serotonin” OR “neuroinflammation” OR “myokines”). Additional condition-specific searches were performed using terms such as “exercise AND fibromyalgia”, “exercise AND chronic low back pain”, “exercise AND knee osteoarthritis”, and “exercise AND whiplash-associated disorders”. Additional articles were identified through reference chaining from key reviews, meta-analyses, and highly relevant mechanistic or clinical studies. Priority was given to peer-reviewed articles, recent reviews, meta-analyses, randomized controlled trials, experimental human studies, and translational animal studies directly relevant to the biological mechanisms discussed. Older studies were included when they provided foundational mechanistic evidence or remained highly relevant to the review’s conceptual framework. Studies were selected based on their relevance to the main domains of this review: neural mechanisms of exercise-induced hypoalgesia, endogenous opioid and endocannabinoid signaling, serotonergic and noradrenergic modulation, neuroimmune and myokine-mediated pathways, musculoskeletal adaptations, interindividual variability, and clinical implications for common chronic pain conditions. The clinical section focused on chronic low back pain, knee osteoarthritis, fibromyalgia, whiplash-associated disorders, and kinesiophobia because these conditions are prevalent, clinically relevant, and frequently discussed in relation to exercise-based pain management. Because this is a narrative review rather than a systematic review or meta-analysis, no formal PRISMA protocol, risk-of-bias assessment, or quantitative evidence grading was performed. Efforts were made to minimize selection bias by including studies across different populations, methodologies, and levels of evidence. The aim was not to provide an exhaustive synthesis of all available studies, but to integrate representative and high-relevance evidence across molecular, cellular, systems-level, and clinical domains. Particular attention was given to distinguishing direct evidence from human exercise-induced hypoalgesia studies, translational evidence from preclinical models, and broader mechanistic inferences from adjacent fields such as stress biology, neuroinflammation, and exercise physiology.

## 2. Exercise-Induced Hypoalgesia and Stress Reduction: Mechanisms from Molecular to Systems Level

To facilitate a structured understanding of these mechanisms, this section is organized across hierarchical biological levels. We first describe systems-level and circuit-based mechanisms of descending pain modulation and stress regulation. This is followed by detailed discussion of key molecular signaling pathways, including opioidergic, endocannabinoid, and serotonergic systems. We then examine neuroimmune and myokine-mediated mechanisms, before addressing musculoskeletal adaptations and interindividual variability. This organization reflects the multidimensional and integrative nature of exercise-induced hypoalgesia and its relationship with stress regulation. Exercise-induced hypoalgesia (EIH) refers to a reduction in pain sensitivity following acute or chronic exercise, commonly observed as increases in pressure pain thresholds, decreased temporal summation, and reduced subjective pain ratings [1,2]. EIH is primarily mediated by activation of descending inhibitory pathways originating in the periaqueductal gray (PAG) and relayed via the rostral ventromedial medulla (RVM) and locus coeruleus to the spinal dorsal horn [3,4]. Descending monoaminergic projections contribute to inhibitory control over nociceptive transmission, while connections with hypothalamic, limbic, and autonomic centers provide a framework through which exercise may influence both pain modulation and stress responsivity [3,4,5,6,7,25]. Within this brainstem circuitry, PAG and RVM neurons are interconnected with hypothalamic, limbic, and autonomic centers, providing an anatomical substrate through which exercise may influence both pain modulation and stress regulation [3,4,5,6,7]. Experimental and clinical data indicate that individuals with chronic musculoskeletal pain often exhibit deficient descending inhibition and exaggerated temporal summation, together with blunted cardiovascular and neuroendocrine adaptation to stressors, and that structured exercise training can partially restore these defects [2,3,7,13,26].

At the molecular level, exercise is associated with activation of endogenous neuromodulatory systems, including opioid, endocannabinoid, serotonergic, and noradrenergic pathways. These systems are presented here as components of a broader pain–stress regulatory network and are discussed in greater detail in the following subsections [5,9,11,12]. From the perspective of stress biology, acute exercise represents a controlled physiological stressor that transiently activates the hypothalamic–pituitary–adrenal (HPA) axis and sympathetic nervous system via corticotropin-releasing hormone-secreting neurons in the paraventricular nucleus, pituitary corticotrophs, and adrenocortical cells that produce glucocorticoids [6,8]. With repeated training, these cell populations undergo regulatory adaptations that strengthen negative feedback through glucocorticoid receptors in the hippocampus, prefrontal cortex, and hypothalamus, which may contribute to more efficient HPA axis regulation and termination of HPA responses, and reduced cortisol reactivity to standardized psychosocial stressors [6,8]. At the same time, exercise-induced changes in autonomic nuclei in the brainstem and intrinsic cardiac neurons support increased vagal efferent activity and higher heart rate variability, an objective index of improved stress resiliency and flexible autonomic control [7]. Meta-analytic evidence indicates that aerobic exercise programs can downregulate circulating cortisol in patients with depression and are associated with clinically relevant decreases in anxiety and perceived stress, which may reflect these underlying cellular and molecular adaptations of the HPA axis and autonomic nervous system [5,6,7].

**Figure 1:**
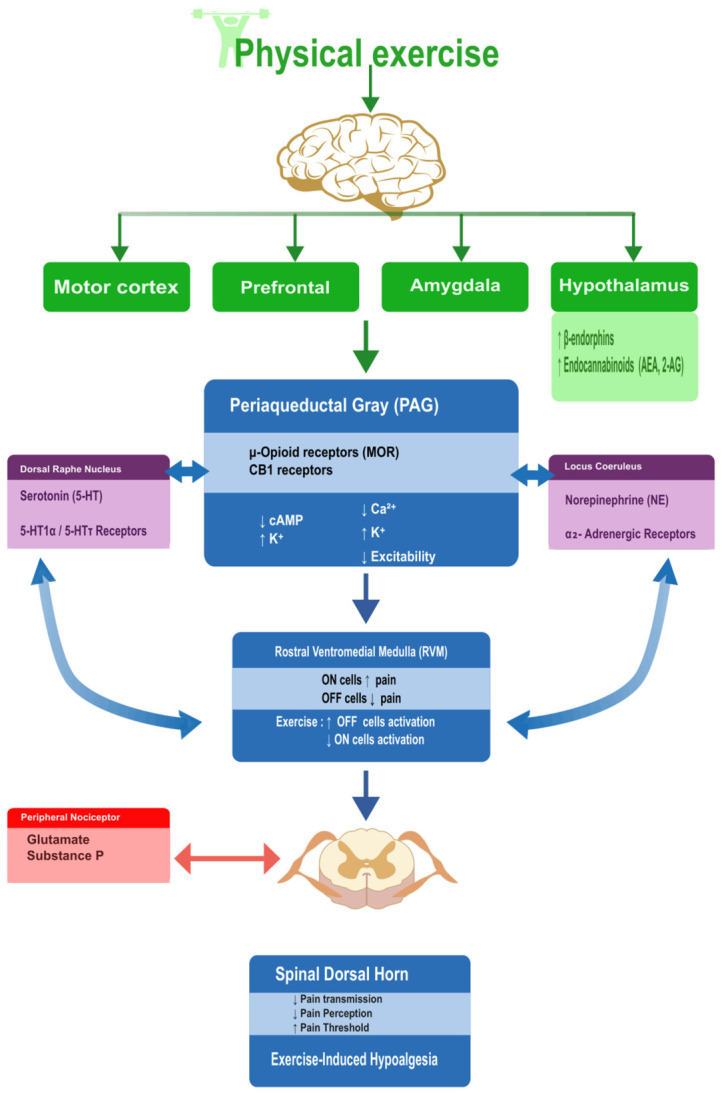
Neurobiological mechanisms of descending pain modulation during exercise. Exercise activates cortical and subcortical regions, including the prefrontal cortex, amygdala, and hypothalamus, leading to increased release of endogenous opioids and endocannabinoids. These systems modulate PAG-RVM pathways, serotonergic and noradrenergic signaling, and spinal nociceptive transmission, resulting in reduced pain perception and increased pain thresholds.

Chronic pain conditions are often associated with impaired descending inhibition and spinal hyperexcitability, linked to altered expression of glutamate receptors such as NMDA and AMPA receptors, reduced GABA-A receptor function, and activation of microglia and astrocytes that release pro-inflammatory mediators, including interleukin-1 beta, tumor necrosis factor-alpha, and brain-derived neurotrophic factor (BDNF) [13]. These glial-derived mediators act through intracellular cascades, including nuclear factor kappa B and mitogen-activated protein kinases, to enhance synaptic efficacy in nociceptive pathways and promote central sensitization [13]. Chronic psychological stress engages overlapping neuroimmune mechanisms, with prolonged glucocorticoid exposure and HPA dysregulation biasing microglia toward a pro-inflammatory phenotype, impairing neurogenesis, and reducing synaptic plasticity in hippocampal and prefrontal circuits that are important for both pain modulation and stress regulation [8,13]. Repeated aerobic or high-intensity exercise induces neuroplastic adaptations that counteract these changes, including increased BDNF expression, activation of PGC-1 alpha-linked transcriptional programs downstream of AMP-activated protein kinase, improved mitochondrial biogenesis, and a shift toward an anti-inflammatory cytokine profile in neurons and glia [4,27,28,29]. These overlapping mechanisms suggest that exercise may support more adaptive nociceptive modulation while also influencing cellular processes involved in stress vulnerability (Figure 1). However, the strength of evidence differs across pathways, with some mechanisms supported directly by human studies and others derived mainly from preclinical or translational models [4,8,13,27,28,29].

Within a biopsychosocial framework, exercise can therefore be understood as a multimodal intervention that links molecular, cellular, and systems-level adaptations. Rather than acting through a single analgesic pathway, physical activity appears to engage overlapping neural, neuroimmune, endocrine, and musculoskeletal processes that may collectively influence pain sensitivity, stress responsivity, emotional regulation, and functional recovery [1,2,3,4,5,6,7,8,12,13,26,27]. In clinical populations with chronic pain and central sensitization features, preserved or enhanced exercise-induced hypoalgesia has been associated with more favorable outcomes, suggesting that the capacity to recruit adaptive pain-modulatory responses may be clinically relevant [26].

### 2.1. Endocannabinoid and Opioid Signaling in Exercise-Induced Modulation of Pain and Stress

Endogenous opioid and endocannabinoid signaling represent two major neuromodulatory systems through which exercise may influence both pain perception and stress-related responses. These pathways are particularly relevant because they operate within overlapping pain-modulatory, limbic, and hypothalamic circuits, linking nociceptive inhibition with affective regulation and stress adaptation [10,11,12,30]. At the systems level, these mechanisms interact with descending pain modulatory pathways and stress-regulatory circuits, linking nociceptive processing with broader neuroendocrine and autonomic responses [31,32]. At the molecular level, human PET evidence suggests that acute high-intensity exercise is associated with endogenous opioid release in frontolimbic and pain-modulatory regions, reflected by reduced mu-opioid receptor (MOR) availability consistent with endogenous ligand binding and receptor internalization [11]. Opioidergic signaling contributes to both sensory and affective components of exercise-induced analgesia [11]. In limbic and hypothalamic nuclei, the same opioid peptides modulate stress-related circuits by dampening HPA axis activation and attenuating negative affective responses to aversive stimuli, suggesting that exercise-driven opioid release is a common mechanism for both hypoalgesia and acute stress relief [11,31]. In parallel, exercise elevates circulating endocannabinoids (AEA and 2-AG), suggesting increased activity of canonical ECS biosynthetic pathways [12,33,34,35]. These systemic changes in AEA and 2-AG levels have been linked to positive affect, reduced tension, and improved stress coping following aerobic exercise, suggesting that ECS activation may contribute to both nociceptive and stress-related effects of physical activity [12,34,35].

Central ECS effects are mediated primarily by CB1 receptors abundantly expressed in PAG, RVM, amygdala, hippocampus, prefrontal cortex, and dorsal horn, where their activation inhibits adenylyl cyclase, decreases cAMP, and suppresses presynaptic glutamate and substance P release. CB1 signaling also engages MAPK and PI3K-Akt cascades that promote synaptic plasticity and activity-dependent remodeling of excitatory and inhibitory synapses [33,36]. Within stress-regulatory circuits, CB1 receptors located on glutamatergic and GABAergic terminals in the amygdala, hippocampus, and hypothalamus constrain excitatory drive onto HPA axis neurons and contribute to rapid negative feedback during and after stress exposure, thereby buffering excessive glucocorticoid release and supporting stress adaptation [33,34,35,37]. Peripherally and in glial cells, CB2 receptor activation downregulates NF-kappaB and MAPK signaling, reduces TNF-alpha and IL-1 beta, and promotes an anti-inflammatory microglial/macrophage phenotype, thereby restoring inhibitory tone in nociceptive circuits and limiting the pro-inflammatory consequences of chronic stress and persistent pain [13,38,39,40]. Preclinical studies show that genetic or pharmacologic disruption of CB1 signaling can abolish exercise-related affective benefits and cutaneous analgesia, supporting an important role for the ECS in exercise-related analgesic and stress-modulating responses [12,34,35,41].

Cannabinoid–opioid cross-talk is well established: both systems couple to Gi/o pathways and interact functionally to support analgesic synergy during noxious modulation [42,43]. Co-localization of MOR and CB1 receptors on overlapping neuronal populations in PAG, RVM, and limbic circuits allows convergent regulation of neuronal excitability and transmitter release, such that concurrent activation by endogenous opioids and endocannabinoids during exercise may amplify descending inhibition and dampen stress reactivity [12,33,42,43]. In addition, endocannabinoid–serotonin interactions modulate dorsal raphe excitability and transmission, providing a mechanistic basis for ECS–monoaminergic integration within descending pain control during exercise [44]. Because dorsal raphe 5-HT projections innervate both spinal nociceptive networks and limbic structures involved in anxiety and stress appraisal, ECS-driven modulation of raphe output has the potential to simultaneously alter pain processing and stress-related behavior [34,44,45,46]. Together, opioidergic, endocannabinoid, and monoaminergic interactions may contribute to reduced nociceptive transmission, modulation of neuroinflammation, and regulation of stress-related circuitry. Exercise may therefore influence pain thresholds, emotional state, and stress responsivity, although the relative contribution of each pathway likely varies across individuals and exercise protocols [5,11,12,33,34,35].

### 2.2. Opioidergic and Serotonergic Modulation of Descending Pain and Stress Circuits: Cellular and Molecular Mechanisms in Exercise-Induced Hypoalgesia

A pivotal molecular axis associated with exercise-induced hypoalgesia (EIH) involves the endogenous opioid system within the periaqueductal gray (PAG)-rostral ventromedial medulla (RVM) circuitry. The PAG is involved in integrating ascending nociceptive input and may recruit descending inhibitory projections through mu-opioid receptor (MOR) activation. MORs are Gi/o-coupled receptors that inhibit adenylyl cyclase, reduce intracellular cAMP, activate G-protein-regulated inwardly rectifying K+ (GIRK) channels, and suppress voltage-gated Ca^2+^ channels [11,47]. This may disinhibit PAG output neurons projecting to the RVM, facilitating descending inhibition of spinal nociceptive transmission [11,31]. The same MOR-expressing networks in PAG, RVM, and hypothalamus interact closely with limbic and hypothalamic nuclei that regulate hypothalamic–pituitary–adrenal (HPA) axis activity, so that endogenous opioids not only shape nociceptive transmission but also modulate stress responsivity and emotional reactivity to aversive stimuli [11].

During exercise, hypothalamic and brainstem nuclei release beta-endorphins and enkephalins that engage MORs in pain-modulatory and limbic regions, potentially attenuating both sensory and affective pain dimensions [2]. Human PET studies show decreased MOR availability after exercise, consistent with endogenous ligand binding and receptor internalization mediated by beta-arrestin-dependent trafficking [11]. Beyond antinociception, endogenous opioid release in the hypothalamus, amygdala, and prefrontal cortex may dampen HPA axis activation and reduces negative affect, indicating that acute exercise-driven opioid signaling may contribute to both hypoalgesia and transient reductions in perceived stress and anxiety [6,48]. MOR activation can further engage MAPK, ERK, and CREB phosphorylation, regulating transcriptional programs associated with neural plasticity and antinociception [49]. These same intracellular cascades influence gene expression in stress-related circuits, providing a molecular substrate through which repeated exercise bouts may contribute to long-term adaptations in stress circuitry [41,49].

Serotonergic signaling synergizes with opioidergic modulation within the RVM and spinal dorsal horn. Receptor-specific effects include antinociception via 5-HT1A and 5-HT7 subtypes and facilitation or hyperalgesia via 5-HT2A, 5-HT2B, and 5-HT3 receptors, depending on context [50,51]. Exercise alters this serotonergic tone: in preclinical models, low- to moderate-intensity exercise reduces serotonin transporter (SERT) expression in brainstem nuclei in preclinical models (including the RVM), prolonging extracellular 5-HT and reinforcing descending inhibition. Pharmacologic opioid blockade attenuates both EIH and these serotonergic adaptations [52,53,54]. In parallel, 5-HT1A and 5-HT7 receptors in the prefrontal cortex and hippocampus are key regulators of mood, anxiety, and stress coping; chronic stress paradigms reduce 5-HT1A receptor expression and signaling in these regions, whereas interventions that enhance serotonergic tone or receptor function mitigate stress-induced behavioral deficits [50,51,55]. Exercise-induced upregulation of serotonergic tone and receptor function in raphe–prefrontal–hippocampal pathways is therefore poised to contribute both to enhanced descending pain inhibition and to improved regulation of stress and anxiety.

At the transcriptional level, repeated exercise elevates brain-derived neurotrophic factor (BDNF) and upregulates serotonergic biosynthetic enzymes such as tryptophan hydroxylase-2 (TPH2) within relevant nuclei, which may support serotonergic output and promote synaptic remodeling [12,56,57]. BDNF signaling via TrkB receptors supports dendritic complexity, synaptic strength, and neurogenesis in the hippocampus and prefrontal cortex, regions crucial for the cognitive and emotional regulation of both pain and stress. Chronic stress and depression are consistently associated with reductions in BDNF expression and impaired trophic support, while voluntary exercise has been shown in preclinical models to reverse some of these changes and enhances resilience to subsequent stressors, in part via coordinated action of BDNF and other trophic factors [56]. Thus, exercise-driven transcriptional adaptations in monoaminergic and neurotrophic systems can be viewed as a shared mechanism that may contribute to sustained EIH and long-term reductions in stress vulnerability.

Conversely, chronic stress and persistent pain activate microglia and astrocytes, elevating IL-1 beta and TNF-alpha levels and impairing MOR and 5-HT1A receptor function via NF-kappaB-dependent mechanisms that drive central sensitization [13]. Pro-inflammatory signaling alters receptor trafficking and G-protein coupling, reducing the efficacy of endogenous opioid and serotonergic inhibitory pathways and thereby amplifying both nociceptive and stress-related neural responses [13,41]. By attenuating neuroinflammation, exercise may help restore more normal receptor function and signaling efficiency, contributing to improved pain modulation and a more adaptive stress response.

Concurrently, aerobic exercise elevates circulating endocannabinoids such as anandamide and 2-arachidonoylglycerol (2-AG), which activate CB1 receptors to inhibit presynaptic glutamate and substance P release and CB2 receptors on immune cells to suppress pro-inflammatory cytokine production [39,40,58]. Pharmacologic or genetic disruption of CB1/CB2 signaling can abolish exercise-related analgesia in animal models, supporting an important contribution of endocannabinoid mechanisms to EIH [41,58]. The endocannabinoid system also appears to play an important role in fear, anxiety, and stress regulation, modulating synaptic transmission within the amygdala, hippocampus, and prefrontal cortex, facilitating fear extinction, and constraining HPA axis activation during and after stress exposure [55]. Exercise-induced elevations in anandamide and 2-AG have been linked to improved mood state, reduced anxiety, and the subjective experience of a runner’s high, suggesting that the same endocannabinoid signals supporting hypoalgesia also mediate acute stress relief and improved emotional regulation [41,55,58].

Together, opioidergic, serotonergic, and endocannabinoid systems may interact to restore descending inhibitory tone, promote adaptive neuroplasticity, and counter maladaptive sensitization processes underlying chronic pain. Because these transmitter systems are also embedded within limbic and hypothalamic networks that regulate HPA axis activity, autonomic outflow, and emotional processing, their exercise-induced modulation provides a mechanistic bridge between EIH and exercise-driven reductions in chronic stress, anxiety, and negative affect [11,41,50,52,54,55,59,60].

### 2.3. Myokine-Driven Anti-Inflammatory and Neuroimmune Mechanisms Linking Exercise, Pain, and Stress

Skeletal muscle functions as an active endocrine organ that releases myokines in response to mechanical and metabolic stimuli during contraction, acting in a paracrine, autocrine, and endocrine manner [14]. Among these, interleukin-6 (IL-6) is a pivotal signaling molecule. During acute exercise, IL-6 is secreted primarily by contracting myofibers via calcium-dependent mechanisms and AMP-activated protein kinase (AMPK)-linked pathways, rather than classical inflammatory triggers such as pathogen-associated signals [15,16]. This release signals through the gp130-associated JAK/STAT3 pathway, inducing transcription of anti-inflammatory cytokines such as interleukin-1 receptor antagonist (IL-1Ra) and interleukin-10 (IL-10), while suppressing NF-kappaB-mediated production of pro-inflammatory mediators including tumor necrosis factor alpha (TNF-alpha) and interleukin-1 beta (IL-1beta) [17]. The net effect is a transient shift toward an anti-inflammatory phenotype both systemically and within the central nervous system [17,61]. Because chronic psychological stress and major depressive disorder are frequently accompanied by low-grade systemic inflammation, microglial activation, and dysregulated hypothalamic–pituitary–adrenal axis activity, repeated exercise-induced activation of this IL-6/IL-10 axis may mitigate stress-related inflammatory load and contribute to improved stress resilience and mood regulation in parallel with its role in hypoalgesia [17,61,62,63].

Beyond IL-6, other exercise-induced myokines such as irisin (cleaved from fibronectin type III domain-containing protein 5, FNDC5) and secreted protein acidic rich in cysteine (SPARC) contribute to tissue homeostasis, neuroprotection, and central modulation of pain and stress [64,65]. Irisin activates the peroxisome proliferator-activated receptor gamma coactivator 1 alpha (PGC-1alpha)-FNDC5/brain-derived neurotrophic factor (BDNF) axis, enhancing mitochondrial biogenesis and reducing oxidative stress in neurons and glia [66]. In rodent and human studies, this axis has also been linked to hippocampal neurogenesis, synaptic remodeling, and antidepressant-like effects of exercise, positioning irisin as a candidate mediator of both pain relief and stress reduction [66,67]. SPARC regulates extracellular matrix turnover and mitochondrial adaptation in skeletal muscle cells, supporting structural remodeling and cellular stress responses associated with physical exercise [65,68]. Through their combined actions, irisin, SPARC, and other myokines participate in a muscle–brain communication axis that targets neuroinflammation, oxidative stress, and synaptic plasticity in pain- and stress-related circuits [18,62,64,65,69].

At the cellular level, regular aerobic training suppresses microglial and astrocytic activation in the dorsal horn, which is a hallmark of chronic pain, with a mechanistic contribution from IL-6/IL-10-driven signaling that limits glial release of TNF-alpha, IL-1beta, and BDNF [61]. Analogous myokine-dependent effects have been reported in supraspinal regions, where reductions in neuroinflammatory markers and restoration of BDNF signaling accompany exercise-induced improvements in cognition and affective behavior [62,63,66,67,69,70]. As a result, exercise attenuates central sensitization and helps restore inhibitory neurotransmission, including normalization of GABAergic and glycinergic tone in the spinal cord [18,62,71], while simultaneously dampening neuroimmune cascades that contribute to stress-related disorders and depression [62,63,67,69].

Genetic variability further modulates these pathways. Polymorphisms in IL-6 (for example, rs1800795, −174G/C), TNF-alpha (−308G/A), and the mu-opioid receptor gene OPRM1 (A118G) have been associated with differences in cytokine production or receptor function, contributing to inter-individual variability in pain sensitivity and in the magnitude of exercise-induced hypoalgesia [72]. Emerging data suggest that similar immunogenetic profiles may shape vulnerability to stress-related mood disorders and the degree to which individuals benefit psychologically from exercise interventions, although this remains an active area of investigation [63,72].

**Figure 2:**
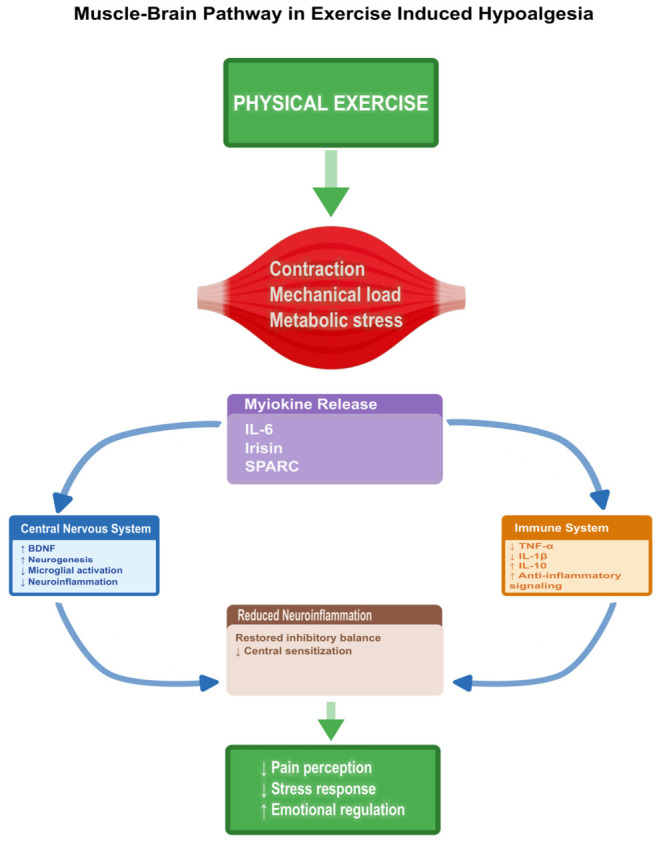
Muscle–brain pathway. Muscle–brain pathway underlying exercise-induced hypoalgesia and stress regulation. Physical exercise induces muscle contraction, mechanical load, and metabolic stress, leading to the release of myokines such as IL-6, irisin, and SPARC. These signals modulate central nervous system and immune responses, promoting anti-inflammatory signaling, reducing neuroinflammation, and restoring inhibitory balance, ultimately decreasing pain perception and stress while improving emotional regulation.

Collectively, the anti-inflammatory and neuroimmune-modulatory effects of exercise-derived myokines constitute a mechanistic bridge between peripheral metabolic activity and central regulation of both pain and stress. By shifting systemic cytokine balance toward an anti-inflammatory phenotype, restraining microglial and astrocytic activation, and promoting BDNF-dependent neuroplasticity in pain- and stress-related networks, myokines help couple skeletal muscle activity to hypoalgesia, stress buffering, and improved emotional well-being. This muscle-brain axis has increasing translational relevance in chronic pain and stress-related disorders [18,62,63,67,69]. From a translational perspective, the muscle–brain axis provides a biologically plausible link between peripheral muscle activity and central pain–stress regulation. By connecting exercise-induced metabolic activity with neuroimmune modulation, this muscle–brain pathway may help explain how sustained physical activity influences inflammatory pain mechanisms, stress-related vulnerability, and emotional regulation [17,61,62]. A schematic overview of the muscle–brain axis linking exercise, neuroinflammation, and pain modulation is presented in Figure 2.

### 2.4. Musculoskeletal Adaptations Linking Exercise, Pain, and Function

At the musculoskeletal level, exercise elicits biomechanical and cellular adaptations that enhance tissue resilience and attenuate nociceptive input. Repeated mechanical loading during aerobic and resistance training activates mechanotransduction cascades in myocytes, tenocytes, and chondrocytes, including focal adhesion kinase (FAK)/mitogen-activated protein kinase (MAPK,ERK1/2) and PI3K-Akt pathways, thereby promoting anabolic signaling, upregulating IGF-1 expression, and stimulating collagen I/III synthesis to enhance connective tissue integrity and joint stability [19,20,21,22]. In skeletal muscle, physiological levels of contraction-induced reactive oxygen species (ROS) act as signaling intermediates that upregulate peroxisome proliferator-activated receptor gamma coactivator 1-alpha (PGC-1alpha), supporting mitochondrial biogenesis, oxidative capacity, and antioxidant defense [23]. In tendons and ligaments, cyclic strain facilitates extracellular matrix remodeling through transforming growth factor beta (TGF-beta)- and scleraxis-dependent mechanisms, maintaining collagen fibril organization and reducing susceptibility to microtrauma [21,22]. These peripheral adaptations optimize load distribution across joints and diminish the release of danger-associated molecular patterns (DAMPs) from stressed tissues, thereby reducing peripheral nociceptor sensitization and downstream central sensitization [24].

Clinically, structured exercise programs improve pain and functional outcomes in common musculoskeletal pain conditions. For chronic low back pain, exercise therapy provides moderate-certainty benefits over minimal care or non-exercise comparators [73], whereas for knee osteoarthritis, multiple modalities including aerobic, resistance, aquatic, yoga, and tai-chi training reduce pain and improve physical function [10]. Within a biopsychosocial framework, restoring movement confidence and physical competence through exercise complements these biological mechanisms, helping to disrupt the pain–disability cycle and reduce fear of movement (kinesiophobia) [74]. These musculoskeletal adaptations complement central and neuroimmune mechanisms by reducing peripheral nociceptive input, improving load tolerance, and supporting functional recovery. In this way, exercise may act not only as a biological modulator of pain and stress, but also as a functional intervention that helps interrupt the pain–disability cycle [73,74].

### 2.5. Interindividual Variability in Exercise-Induced Hypoalgesia

Interindividual variability in exercise-induced hypoalgesia (EIH) reflects converging genetic, epigenetic, neurochemical, and psychosocial factors. Human studies consistently reveal substantial between-subject differences in the magnitude of EIH and in the functional integrity of descending inhibitory pathways, even under standardized exercise protocols [10]. Genetic variability within immune and opioid systems contributes to interindividual heterogeneity. For example, the IL6 −174G/C promoter polymorphism has been associated with variability in circulating interleukin-6 responses to exercise, indicating genotype-dependent inflammatory reactivity to physical stressors [75]. Similarly, gene–physical activity interaction studies involving TNF promoter variants (for example rs1800629/−308 G>A) suggest genotype-dependent physiological responses to habitual physical activity [76]. In the endogenous opioid system, the OPRM1 A118G (rs1799971) variant is associated with variability in conditioned pain modulation and interacts with serotonergic polymorphisms to shape endogenous analgesia [77,78,79]. In fibromyalgia cohorts, such gene–gene interactions between OPRM1 and serotonergic genes have been linked to differences in endogenous pain inhibitory capacity, even though single polymorphisms alone account for only a small portion of the clinical phenotype [77,78]. Functionally, A118G alters mu-opioid receptor binding properties and availability, providing a plausible molecular basis for interindividual differences in analgesic capacity [79]. Beyond genetic polymorphisms, epigenetic plasticity further modulates responsiveness to exercise. Both single and repeated exercise bouts can reshape DNA methylation and histone marks in genes regulating neuroplasticity and energy metabolism. Notably, exercise induces promoter hypomethylation and upregulated expression of PGC-1alpha in skeletal muscle, as well as epigenetic modulation of BDNF and other plasticity-related genes in the brain [80,81]. Individuals exhibiting blunted exercise-induced epigenetic remodeling may consequently experience smaller analgesic and mood-related benefits. Phenotypic distinctions also play a role: nociceptive, neuropathic, and centralized pain states differ in their neuroimmune profiles. Centralized pain conditions exhibit pronounced neuroinflammation and impaired descending inhibition, which helps explain why extended or multimodal exercise programs are often required to restore inhibitory tone and elicit EIH in clinical populations [10,13].

Looking forward, the emerging field of precision exercise medicine seeks to stratify responders using integrated multi-omics approaches, including transcriptomics, proteomics, and metabolomics, combined with objective biomarkers of endogenous pain modulation [82,83]. This framework aims to individualize exercise prescriptions in terms of modality, intensity, and timing, optimizing analgesic efficacy—and potentially stress-buffering effects—according to each person’s molecular and physiological signature. This variability underscores the need to move beyond uniform exercise prescriptions toward mechanism-informed and phenotype-sensitive approaches. Integrating clinical presentation with biomarkers of pain modulation, inflammation, stress physiology, and exercise tolerance may help identify which patients are most likely to benefit from specific exercise strategies [10,82,83].

## 3. Structured Exercise and Clinically Informed Prescription in Chronic Pain

Structured exercise is a clinically supported component of chronic pain management, with effects that extend beyond general improvements in physical fitness. Exercise interventions can influence pain intensity, disability, physical function, kinesiophobia, and experimental pain modulation, although the magnitude of these effects varies across patient populations and exercise protocols. These clinical outcomes are supported by underlying neurobiological mechanisms, including activation of descending inhibitory pathways, engagement of endogenous opioid and endocannabinoid systems, modulation of monoaminergic signaling, and attenuation of neuroinflammation [2,3,4,9,12,13].

Large-scale synthesized evidence in chronic non-specific low back pain, including 249 randomized trials with a total of 24,486 adult participants, demonstrates that exercise is superior to no treatment, usual care, or placebo for improving pain and functional outcomes [84]. Across these trials, exercise interventions were associated with small-to-moderate effect sizes for pain reduction (standardized mean difference approximately −0.3 to −0.5) and clinically meaningful improvements in disability. Adverse events were generally minor and transient, most commonly involving short-term pain exacerbation or muscle soreness [84].

### 3.1. Graded Initiation and Symptom-Contingent Progression

Clinical application may be more effective when guided by patient phenotype rather than fixed exercise templates. Individuals presenting with central sensitization, high pain irritability, fatigue, or fear of movement may benefit from low-intensity, graded exercise with gradual progression [10,85]. Evidence on exercise-induced hypoalgesia (EIH), derived from both healthy individuals and patients with chronic musculoskeletal pain, suggests that clinically meaningful analgesic effects are often observed after repeated exposure over 8–12 weeks, with increases in pressure pain thresholds of approximately 10–20% and reductions in temporal summation [10,85]. However, patients with chronic pain frequently demonstrate blunted or absent acute hypoalgesic responses, reflecting impaired descending inhibitory function [85]. Experimental studies further show that low-to-moderate intensity aerobic or resistance exercise can produce acute increases in pain thresholds of approximately 5–15%, depending on exercise intensity and population characteristics [85].

### 3.2. Chronic Low Back Pain: Structured Exercise Improves Pain and Disability

Participants in these trials were typically adults aged 30–65 years with chronic non-specific low back pain persisting for more than 3 months, often presenting with reduced physical activity levels, moderate functional impairment, and baseline pain scores of approximately VAS ≥ 4–6. Randomized controlled trials demonstrate that structured exercise interventions, including Pilates-based programs performed 2–3 times per week over 8–12 weeks, result in reductions in pain intensity of approximately 1.5–3.0 points on a 10-point VAS scale and improvements in disability scores of approximately 10–20% compared with minimal intervention or education alone [86,87]. These improvements are clinically meaningful and align with enhanced descending inhibitory control, reduced spinal excitability, and attenuation of neuroimmune activation described in mechanistic studies [2,3,4,13].

### 3.3. Knee Osteoarthritis: Modality Selection Can Be Flexible

Study populations primarily consisted of older adults aged 60–75 years with radiographically confirmed knee osteoarthritis, moderate pain severity, reduced mobility, and frequent comorbidities such as overweight or obesity. Randomized controlled trials show that tai chi interventions performed 2–3 times weekly over approximately 12 weeks lead to reductions in WOMAC pain scores of approximately 20–30% and improvements in physical function of similar magnitude [88,89]. In comparative trials, tai chi demonstrated comparable improvements to conventional physical therapy, with additional benefits in psychosocial outcomes, including reduced perceived stress and improved quality of life [89].

### 3.4. Fibromyalgia: Supervised and Progressive Exercise Is Critical

Participants in these trials were predominantly middle-aged women (40–60 years) with widespread pain, fatigue, sleep disturbances, and reduced exercise tolerance, reflecting the typical clinical profile of fibromyalgia. Randomized controlled trials indicate that supervised aerobic and resistance training programs conducted over 8–16 weeks can produce reductions in pain scores of approximately 15–30%, alongside improvements in quality of life and depressive symptoms [90]. Exercise protocols typically include low-to-moderate intensity aerobic training combined with progressive resistance exercises, emphasizing gradual progression and supervision to improve adherence and minimize symptom exacerbation.

### 3.5. Chronic Whiplash-Associated Disorders: Altered Hypoalgesic Responses

Study cohorts included individuals aged 20–60 years with chronic whiplash-associated disorders and persistent symptoms lasting several months to years following cervical trauma, often characterized by central sensitization and heightened pain sensitivity. Experimental studies show that aerobic exercise often fails to produce significant increases in pain thresholds, whereas isometric exercise may elicit modest hypoalgesic responses [26,91]. Quantitative sensory testing further demonstrates reduced conditioned pain modulation, consistent with dysfunction in descending inhibitory pathways [26,91].

### 3.6. Kinesiophobia and Biopsychosocial Integration

Populations examined in these studies included individuals with chronic musculoskeletal pain characterized by elevated fear-avoidance beliefs, reduced movement confidence, and avoidance of physical activity. Systematic reviews indicate that exercise interventions incorporating graded exposure can lead to reductions in kinesiophobia scores of approximately 10–20%, alongside improvements in functional outcomes and daily activity participation [74]. These effects reflect both cognitive–behavioral adaptations and normalization of neural circuits involved in threat processing and emotional regulation [5,7].

### 3.7. Practical Principles for Clinically Informed Exercise Prescription

Current evidence supports a shift from generic exercise recommendations toward a more structured and individualized prescription model. Regular and repeated exercise appears to be an important determinant of analgesic adaptation, with cumulative exposure over 8–12 weeks or longer required for sustained effects [2,3,4,13]. When tolerated, moderate-to-vigorous intensity exercise may elicit stronger hypoalgesic responses, potentially producing greater increases in pain thresholds and larger reductions in pain intensity, although tolerability and symptom stability remain central considerations [9,12].

Exercise type, intensity, and progression should therefore be tailored to patient phenotype, symptom irritability, conditioning level, and psychosocial context. Outcomes should be evaluated using multidimensional measures, including pain intensity, functional capacity, kinesiophobia, and adherence [74,84,85]. Variability in clinical response likely reflects differences in endogenous pain modulation capacity, neuroimmune state, and psychosocial factors [10,84]. Table 1 provides an illustrative overview of representative exercise interventions and associated clinical outcomes across selected chronic pain conditions. To improve clarity and readability, key clinical findings discussed in the text are summarized in the table. The included examples reflect commonly studied conditions and typical clinical responses rather than a comprehensive or quantitative comparison, and the table should not be interpreted as a systematic synthesis of evidence.

## 4. Conclusions

Exercise-induced hypoalgesia reflects the integration of neural, neuroimmune, and musculoskeletal mechanisms that collectively influence both pain modulation and stress regulation [2,3,4,5,6,7,8,9,10,11,12,13]. Converging evidence suggests that exercise engages descending inhibitory pathways, modulates endogenous opioid and endocannabinoid signaling, and influences inflammatory and neurotrophic processes, thereby contributing to reductions in pain sensitivity and improvements in stress-related physiological and psychological responses [4,5,6,7,8,9,11,12]. At the clinical level, structured exercise interventions are associated with improvements in pain, function, and psychosocial outcomes across multiple chronic pain conditions [1,10,26,73,85,86,87,88,89,90,91]. However, the magnitude and consistency of these effects vary substantially, highlighting the importance of individual factors such as baseline pain mechanisms, neuroimmune state, exercise tolerance, and psychosocial context [10,72,75,76,77,78,79,80,81,82,83]. Several important knowledge gaps remain. Future human studies should integrate mechanistic biomarkers, pain-modulation phenotyping, and clinically meaningful exercise–response profiles to clarify which patients benefit most from specific exercise modalities, intensities, and progression strategies [10,82,83]. Such approaches may support the development of more precise, mechanism-informed, and personalized exercise strategies for chronic pain management and stress-related disorders.

## Figures and Tables

**Table 1:** Illustrative summary of exercise interventions and associated clinical outcomes across selected chronic pain conditions.

Condition	Population	Exercise Protocol	Duration	Outcomes	Key Clinical Notes	References
Chronic low back pain	Adults (30–65), pain > 3 months, VAS ≥ 4–6	Pilates, aerobic, strengthening	8–12 weeks	↓ pain (VAS −1.5 to −3.0), ↓ disability (10–20%)	No single superior modality	[84,88,89]
Knee osteoarthritis	Older adults (60–75), OA, reduced mobility	Tai chi, aerobic, strengthening	~12 weeks	↓ WOMAC pain (20–30%), ↑ function	Low-impact + psychosocial benefits	[86,87]
Fibromyalgia	Middle-aged women (40–60), widespread pain	Aerobic + resistance (supervised)	8–16 weeks	↓ pain (15–30%), ↑ QoL	Supervision + gradual progression	[90]
Whiplash	Adults (20–60), chronic symptoms	Isometric ± aerobic	Variable	Limited EIH, modest effect	Impaired descending inhibition	[12,91]
Kinesiophobia	Chronic pain, fear-avoidance	Graded exposure + exercise	Several weeks	↓ kinesiophobia (10–20%)	Combine physical + behavioral	[67]

Note: ↓ indicates a decrease; ↑ indicates an increase. The table provides an illustrative overview and should not be interpreted as a systematic synthesis of evidence. Values shown are representative ranges from selected cited studies and should not be interpreted as pooled effect estimates or as a quantitative comparison across conditions.

## Data Availability

No new data were created or analyzed in this study.

## Abbreviations

The following abbreviations are used in this manuscript:

2-AG	2-arachidonoylglycerol
5-HT	serotonin
AEA	anandamide
AMPA	alpha-amino-3-hydroxy-5-methyl-4-isoxazolepropionic acid
AMPK	AMP-activated protein kinase
BDNF	brain-derived neurotrophic factor
CB1	cannabinoid receptor type 1
CB2	cannabinoid receptor type 2
DAMPs	damage-associated molecular patterns
ECS	endocannabinoid system
EIH	exercise-induced hypoalgesia
ERK	extracellular signal-regulated kinase
FAK	focal adhesion kinase
FNDC5	fibronectin type III domain-containing protein 5
GABA	gamma-aminobutyric acid
GIRK	G-protein-regulated inwardly rectifying potassium channel
HPA	hypothalamic–pituitary–adrenal
IL-1Ra	interleukin-1 receptor antagonist
IL-6	interleukin-6
IL-10	interleukin-10
MAPK	mitogen-activated protein kinase
MOR	mu-opioid receptor
NE	norepinephrine
NF-kappaB	nuclear factor kappa B
NMDA	N-methyl-D-aspartate
OPRM1	opioid receptor mu 1
PAG	periaqueductal gray
PGC-1alpha	peroxisome proliferator-activated receptor gamma coactivator 1-alpha
PI3K	phosphoinositide 3-kinase
ROS	reactive oxygen species
RVM	rostral ventromedial medulla
SERT	serotonin transporter
SPARC	secreted protein acidic and rich in cysteine
STAT3	signal transducer and activator of transcription 3
TGF-beta	transforming growth factor beta
TNF-alpha	tumor necrosis factor alpha
TPH2	tryptophan hydroxylase 2
VAS	visual analog scale
WOMAC	Western Ontario and McMaster Universities Osteoarthritis Index
